# A Useful SNP Panel to Distinguish Two Cockle Species, *Cerastoderma edule* and *C. glaucum*, Co-Occurring in Some European Beds, and Their Putative Hybrids

**DOI:** 10.3390/genes10100760

**Published:** 2019-09-27

**Authors:** Francesco Maroso, Celia Pérez de Gracia, David Iglesias, Asunción Cao, Seila Díaz, Antonio Villalba, Manuel Vera, Paulino Martínez

**Affiliations:** 1 Department of Zoology, Genetics and Physical Anthropology, ACUIGEN Group, Faculty of Veterinary, Universidade de Santiago de Compostela, Campus of Lugo, 27002 Lugo, Spain; francesco.maroso@gmail.com (F.M.); celia_dac4@hotmail.com (C.P.d.G.); paulino.martinez@usc.es (P.M.); 2 Centro de Investigacións Mariñas (CIMA), Consellería do Mar, Xunta de Galicia, 36620 Vilanova de Arousa, Spain; david.iglesias.estepa@xunta.gal (D.I.); asun@cimacoron.org (A.C.); antonio.villalba.garcia@xunta.gal (A.V.); 3 Department of Zoology, Genetics and Physical Anthropology, Mobile Genomes and Disease Group, CIMUS, Universidade de Santiago de Compostela, Campus of Santiago de Compostela, 15706 Santiago de Compostela, Spain; seiladiazcostas@gmail.com; 4 Department of Life Sciences, University of Alcalá, 28871 Alcalá de Henares, Spain; 5 Research Centre for Experimental Marine Biology and Biotechnology (PIE), University of the Basque Country (UPV/EHU), 48620 Plentzia, Basque Country, Spain; 6 Instituto de Acuicultura, Universidade de Santiago de Compostela, 15705 Santiago de Compostela, Spain

**Keywords:** 2b–RAD, cockles, diagnostic SNPs, hybrids, New Generation Sequencing (NGS), SNaPshot, wildlife forensic

## Abstract

Cockles are highly appreciated mollusks and provide important services in coastal areas. The two European species, edible (*Cerastoderma edule*) and lagoon (*Cerastoderma glaucum*) cockles, are not easily distinguishable, especially when young. Interestingly, the species show different resistance to *Marteilia cochillia*, the parasite responsible for marteiliosis outbreaks, which is devastating cockle production in some areas. *C. edule* is severely affected by the parasite, while *C. glaucum* seems to be resistant, although underlying reasons are still unknown. Hybrids between both species might be interesting to introgress allelic variants responsible for tolerance, either naturally or through artificial selection, from lagoon into edible cockle. Here, we used 2b restriction site-associated DNA sequencing (2b–RAD) to identify single nucleotide polymorphisms (SNP) diagnostic for cockle discrimination (fixed for alternative allelic variants). Among the nine diagnostic SNPs selected, seven were validated using a SNaPshot assay in samples covering most of the distribution range of both species. The validated SNPs were used to check cockles that were suggested to be hybrids by a claimed diagnostic tool based on the internal transcribed spacers of the ribosomal RNA. Although these were shown to be false positives, we cannot rule out the fact that hybrids can occur and be viable. The SNP tool here developed will be valuable for their identification and management.

## 1. Introduction

True cockles are members of the genus *Cerastoderma*, a group of bivalve mollusks widely distributed in the Mediterranean and Atlantic coasts of Europe [1]. Production of cockles, especially the species most valued as food, edible cockle (*Cerastoderma edule* (Linnaeus, 1758)), is especially relevant in Spain, the British Isles, and France, with production reaching up to 24,626 tons in 2017 in Europe [2]. European production of cockles is mostly exported to Galicia (NW Spain), where the species is marketed as fresh or canned food. In addition to food provisioning, cockles provide many ecosystem services [3], which are especially important for the development and support of many coastal areas. Edible cockle production has been severely affected by marteiliosis outbreaks caused by *Martelia cochillia*, especially in Galicia (NW Spain), where the parasite has devastated its production in the Southern estuaries (Rias). This parasite was first recorded in that area in 2012 [4], but its first description was reported in Catalonia (NE Spain) from cockles identified as *C. edule* [5], despite the typical cockle species occurring in the Mediterranean coasts is the lagoon cockle (*Cerastoderma glaucum* (Poiret, 1789)) [1]. Interestingly, in the Galician coast, only *C. edule* is affected by martelliosis, while *C. glaucum* seems to be resistant to this parasite [6]. The known geographic ranges of both *Cerastoderma* species [1] supports the fact that cockles occurring in the Mediterranean Sea are *C. glaucum*, and accordingly, the cockles infected with *M. cochillia* in Catalonia should be *C. glaucum*. This would suggest different susceptibility to this parasite between the Mediterranean (Catalonian) and the Atlantic (Galician) types of *C. glaucum* [6], thus adding new data to the convenience of splitting *C. glaucum* into different species or subspecies [7,8]. This information would refine the previous report suggesting that Catalonian and Galician populations would be included in the same lineage of the three identified for *C. glaucum* using restriction site-associated DNA Sequencing (RADseq) [9], which is widely distributed from the Western Mediterranean to the Baltic Sea [9]. Alternatively, the hypothesis of different virulence between the parasites *M. cochillia* occurring in Catalonia and Galicia should not be discarded. The reasons behind this differential resistance between *C. edule* and *C. glaucum* in Galicia are still unknown, and deserve special attention within the COCKLES EU project following a genomic approach in the most affected area, the Ria of Arousa (42°30′ N–08°56′ W, EAPA_458/2016). Furthermore, the tolerance of *C. glaucum* to *M. cochillia* makes putative hybrids especially interesting. Checking its viability and reproduction would be an interesting way to introgress allelic variants responsible for tolerance either naturally or through artificial selection in *C. edule*.

The two recognized cockle species, *C. edule* and *C. glaucum*, are not easy distinguishable at a glance, especially when they are young. However, there are some morphological characteristics that trained eyes can use for discrimination [10,11]. Hybrids have been claimed to be generated in captivity [12], but limited evidence that they were real hybrids was provided. Despite the two species showing differences in habitat preferences and lifestyle (see Tarnowska et al. [13] for a detailed description), they present overlapping geographical distributions in various sites of the European Atlantic coast [10,11], and overlapping spawning periods have been supported for these species [14]. Although no records of hybrids have been reported to date, their presence has been recently suggested (Seila Díaz, unpublished data) using a reported diagnostic tool based on the rDNA internal transcribed spacer (ITS) [15]. ITSs have been used to discriminate species quite frequently among parasites [16], but also for aquaculture species such as cockles [17] and mud crabs (*Scilla* sp.) [18]. ITSs represent a very useful and straightforward way to develop molecular tools for species discrimination because of the existence of conserved regions for universal primer design in the adjacent 18S, 5.8S, and 28S genes. However, the tandem constitution of rDNA genes and the multiple location and site polymorphism reported for Nucleolar Organizer Regions (NOR) reported in several species, including cockles [19], suggest caution and strict validation using consistent reference tools.

The development of cutting-edge genomic technologies and protocols in the last decade has opened the possibility to develop a wide range of genotyping by sequencing methods that enable simultaneously identifying and genotyping thousands of single nucleotide polymorphisms (SNP) at low cost [20]. This approach has also been applied to compare large portions of the genomes of individuals from closely related species to look for diagnostic markers that make possible suitable discrimination between taxa [21]. Furthermore, these SNP-based tools enable distinguishing F1, F2, and backcrosses with high confidence, thus allowing a refined evaluation of the hybridization degree of individuals and populations. Here, we describe the identification of a set of diagnostic SNPs for a reliable identification of *C. edule*, *C. glaucum*, and their putative hybrids in natural beds of the Atlantic Area, where geographical distribution of both species overlaps. Nine markers fixed for alternative allelic variants identified in “pure” individuals of the two species were validated in samples covering a wide distribution range and tuned up in a SNaPshot assay designed to provide a cheap and straightforward diagnostic tool. This SNP panel will be useful for management of extensive and intensive production areas where both species co-occur, and to address more sophisticated tools for studying the resistance of cockles to marteiliosis.

## 2. Materials and Methods

### 2.1. Samples for Genomic 2b Restriction Site-Associated DNA Sequencing (2b–RAD) Libraries

**Figure 1:**
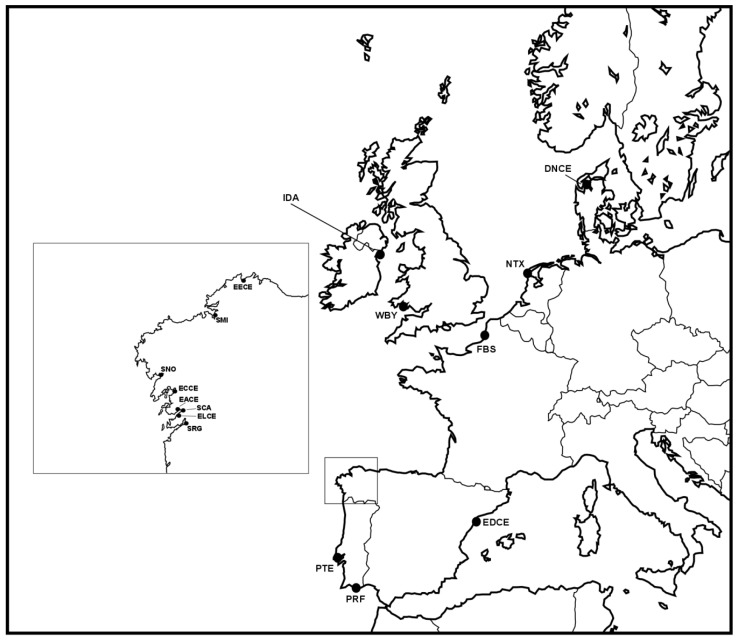
Geographical situation of the natural beds analyzed in the present studies. Codes are shown on Table 1.

Thirty individuals of *C. glaucum* and 120 individuals of *C. edule* collected over 2017–2018 were genotyped using the 2b–RAD genotyping-by-sequencing (GBS) method [22] to identify diagnostic SNPs to distinguish the two species and their hybrids. Edible cockles were collected in four Atlantic European locations: Somme Bay, Miño, Campelo, and Ria Formosa (from north to south, 30 individuals collected in each sampling site) covering a wide distribution range (Table 1; Figure 1), while lagoon cockles were collected in Redondela (N = 30) (Figure 1).

### 2.2. DNA Extraction and Single Nucleotide Polymorphisms Calling and Genotyping

DNA was extracted from gill tissue using the E.Z.N.A. E-96 mollusk DNA kit (OMEGA Bio-tech, Norcross, GA, USA), following the manufacturer recommendations. SNP identification and selection, as well as genotyping and validation protocols, were similar to those described by Maroso et al. [21]. Briefly, genomic DNA was cut using the AlfI IIb restriction enzyme (RE), and then 2b–RAD libraries were constructed by joining adaptors to both fragment ends, followed by PCR amplification using primers targeting specific regions within adaptors. After PCR amplification, DNA from barcoded individuals was pooled for sequencing in an Illumina Next-seq machine. Ninety individuals were routinely multiplexed per run. After demultiplexing and filtering raw reads by quality (Phred value > 30) and the presence of the AlfI restriction site, Stacks 2.0 [23] was used to align all reads from both species to identify and characterize shared genomic loci (RAD–tags) between both species; this information was used to create a consensus catalog of loci and to call SNPs using the whole population data from both species using a de novo approach. A minimum of three identical reads was required, and up to three mismatches were allowed between reads to be considered as part of the same locus. The obtained SNP dataset was parsed to identify diagnostic SNPs (those with different alternative alleles fixed in the two analyzed species). This list was additionally filtered, and RAD–tags containing only one SNP and called in at least 15 *C. glaucum* and 30 *C. edule* specimens were retained. RAD–tags of this filtered dataset were then aligned against the *C. edule* draft genome (Tubío et al, unpublished) and only those aligning to a single genome site were retained. Sequences of the *C. edule* genome 150 bp up- and down-stream from the diagnostic SNP position were used to design external primers using the software Primer3 [24].

### 2.3. Validation of Diagnostic Single Nucleotide Polymorphisms

Among the diagnostic loci retained, a small subset of 20 was selected for the final validation (see Results and Discussion). For this, external primers were checked in three *C. glaucum* and three *C. edule* specimens. Loci were amplified in 15 μL volume, including 1 μL template DNA (~30 ng), 1X PCR Gold Buffer (Applied Biosystems, Forest City, CA, USA), 1.5 mM of MgCl_2_, 100 µM of dNTP, 10 pmol of both forward and reverse PCR primers, and 0.5 U of Amplitaq Gold^TM^ DNA polymerase (Applied Biosystems). Thermal cycling was conducted on a Verity^TM^ 96-Well Thermal Cycler (Applied Biosystems) as follows: initial denaturation at 95 °C for 10 min, 35 cycles of denaturation at 94 °C for 45 s, annealing at 60 °C for 50 s, and extension at 72 °C for 50 s. There was a final extension step at 72 °C for 10 min. Only primer pairs showing a single neat band in agarose gels and the expected amplicon size in both species were selected for further steps. Selected RAD–tag amplicons were sequenced in three *C. glaucum* and three *C. edule* specimens following the ABI Prism BigDye™ Terminator v3.1 Cycle Sequencing Kit protocol in an ABI 3730xl DNA Genetic Analyzer (Applied Biosystems) in order to confirm the consensus sequences around the diagnostic SNPs, from which internal primers could be designed for genotyping. Annotation of these sequences was carried out using BLASTn with default parameters and e-value <1e–5 within the NCBI database. Selected SNPs were genotyped with the SNaPshot methodology (Applied Biosystems), which is based on the fluorescent detection of the SNP variants through a two-step reaction protocol. The first step involves the amplification by PCR of a region including the selected SNP (using the external primers), while the second step is a 1-base sequencing reaction from the adjacent SNP primer (i.e., internal primer). For SNP multiplexing, internal primers were designed with length differences by adding CAGT tails to the 5’ end of the primer sequence (see Table 2). Primer compatibility for multiplexing was checked using ThermoFisher web application [25] and one multiplex reaction following the methodology described by Vera et al. [26] was finally designed to analyze the nine selected SNPs (Table 2). For genotyping, 0.2 µM of each primer was used, both for PCR amplification with the external primers and for the SNaPshot reaction using internal primers. The annealing temperatures for PCR amplification and the SNaPshot reaction were 60 °C and 55 °C, respectively. The obtained SNaPshot assay was finally tested on 48 individuals morphologically identified of each species (total = 96), covering their main distribution ranges and including specimens previously genotyped by 2b–RAD GBS methodology (see Table 1). Finally, five different beds from Denmark (1) and Galicia (4) (total individuals = 46; see Table 1), where both species co-occur and suspected hybrids had been identified (Díaz et al. unpublished data) using the methodology described by Freire et al. [15], were analyzed with the developed SNP tool.

## 3. Results and Discussion

All specimens of both species used for identifying diagnostic SNPs were retained for further analysis, except one *C. glaucum* sample, which was eliminated before filtering due to the low number of raw reads. On average, 2,768,161 reads per specimen were retained after quality filtering. Stacks analysis identified a total of 13,715 SNPs in RAD–tags, including reads from both species, 2057 diagnostic and 11,658 restricted to one or other of the two species (Tables S1 and S2, respectively). Among the latter, 1658 were private of *C. edule* (1190 with a minimum allele frequency (MAF) ≥ 0.05; 71.8%) and 10,000 of *C. glaucum* (3028 with MAF ≥ 0.05; 30.3%). These figures, despite being representative of a particular fraction of the genome (RAD–tags including diagnostic SNPs), strongly suggest a much higher genetic diversity in *C. glaucum* than in *C. edule*, especially regarding the number of SNPs per physical length; furthermore, much of the difference was due to the higher proportion of rare alleles in *C. glaucum* than in *C. edule* (MAF < 0.05: 69.7% vs. 28.2%, respectively), suggestive of higher effective population size and/or no recent bottlenecks. Moreover, the most representative *C. glaucum* beds were located within the Southern glacial refugia of the species (i.e., Iberian Peninsula), where higher genetic diversity is expected [13]. As usually reported in mollusk species [27,28], a global significant heterozygote deficit was observed in both species (F_IS_ > 0; *P* = 0), but was significantly higher in *C. glaucum* than in *C. edule* (F_IS_ 0.252 vs. 0.129).

The 2057 diagnostic loci were identified in 1534 different RAD–tags, meaning that more than one diagnostic SNP was detected in many RAD–tags; nearly 50% contained two diagnostic loci and six RAD–tags up to three diagnostic SNPs (Table S1). Only 157 RAD–tags contained a single diagnostic SNP and no other polymorphism (i.e., private SNPs). Since these 157 RAD–tags showed no genetic variation within species, they were considered the most suitable ones for designing primers, taking the *C. edule* genome as a reference. After eliminating RAD–tags shared by a low number of individuals of the two species, 60 were retained, and among these, 46 matched to unique positions in the *C. edule* genome (Tubío et al., unpublished data). A final selection of 20 RAD–tags was randomly made for the final validation step using the SNaPshot protocol, but the remaining 26 RAD–tags represent a useful repository for increasing the number of diagnostic SNPs if necessary.

Twenty primer pairs were designed with Primer 3 using the *C. edule* genome as a reference and their performance for PCR amplification was checked on agarose gels in three *C. edule* individuals from Ría de Noia (SNO) and three *C. glaucum* from Redondela (SRG). Nine loci showed a single band of expected size, thus being considered suitable for the last step of validation using the SNaPshot methodology (Cerast_31, Cerast_450, Cerast_456, Cerast_586, Cerast_1173A, Cerast_1255, Cerast_1316, Cerast_1400, and Cerast_2530, Table 2). The sequences of these nine RAD–tags confirmed that those markers showed a single diagnostic difference between both species (GenBank Accession Numbers: MN178488–MN178496). Four out of the nine sequences matched against annotated genes, but these markers were not associated with either mtDNA or ribosomal genes (Table S1). Internal primers were then designed adjacent to the SNP position for these nine loci to achieve the best compatibility for multiplexing the SNaPshot mini-sequencing reaction (Table 2). The obtained genotypes in the individuals analyzed with both techniques (i.e., 2b–RAD GBS and SNaPshot) were identical (Table S3), confirming the correspondence between them and giving additional support to the designed molecular tool. When individuals from distant beds not included in the initial RADseq screening were genotyped, diagnostic differences remained for all loci except for Cerast_586 and Cerast_1173A, which showed a low polymorphism for *C. glaucum* in Mediterranean samples (Table S3). Previous molecular tools have been reported for the identification of cockle species, but they were focused on maternally inherited mtDNA [29], on dominant random amplified polymorphic DNA (RAPDs) patterns [30] and on the ITSs of the tandemly arranged rDNA gene family [15,31], all of them displaying different limitations for the identification of hybrids. The seven SNPs here reported as diagnostic and developed by Next Generation Sequencing (NGS) and Genotyping-By-Sequencing (GBS) methodologies allow for a more refined analysis of hybridization between both species if it exists, detecting with fully confidence F1 hybrids and with reasonable accuracy their backcrosses (BC) and F2 hybrids (*P* > 0.95).

The 10 suspected hybrids detected (Díaz et al., unpublished data) using the methodology ITS region [15] were analyzed with the tool here described, and all of them showed pure multilocus genotypes either for *C. edule* (three individuals) or *C. glaucum* (seven individuals) (Table S4), strongly suggesting that they are not hybrids. In fact, the most intense ITS band shown on agarose gels by these individuals corresponded with the assigned species with the SNP tool (data not shown). Although we cannot fully discard a recurrent backcross introgression from one species into another occurring several generations ago, a more plausible explanation for this observation is the existence of an intraspecific rDNA polymorphism in cockles, as previously reported in *C. glaucum* [19] and in many other species [32,33,34,35]. As outlined above, the probability of being a BC or F2 hybrid with pure species genotype would be < 0.05 for seven diagnostic loci, and this would be much lower for the multiple individuals detected showing that pattern. Despite the fact that this observation does not fully invalidate the ITS tool by Freire et al. [15], methodological modifications (e.g., changes in annealing temperature) should be addressed to avoid the appearance of unspecific bands in the gels.

In summary, our data support the usefulness of the seven diagnostic SNPs reported from a large sample of individuals covering the main distribution range for discriminating edible and lagoon cockles; furthermore, a larger SNP diagnostic repository is available in case more markers were needed depending on the goals of the study. In addition, our study highlights the usefulness of NGS strategies to identify diagnostic differences in the genomes of closely related species for the analysis of hybridization in the wild, useful for management of genetic resources in productive areas or in those areas where cockles provide ecosystem services.

## Figures and Tables

**Table 1:** Cockle beds used in the present study. Individuals used for genomic libraries for 2b restriction site-associated DNA sequencing genotyping by sequencing (2b–RAD GBS) and those analyzed with the SNaPshot molecular tool (SNaPshot) developed in the present study are shown.

Bed (Drainage)	Code	Geographical Coordinates	N	Specimens			Technique	
				*Cerastoderma edule*	*Cerastoderma glaucum*	Suspected Hybrids	2bRAD GBS	SNaPshot
Texel—Netherlands (Atlantic)	NTX	53°00.22′ N, 4°46.23′ E	8	8				8
Somme Bay—France (Atlantic)	FBS	50°12.08′ N, 1°37.62′ W	30	30			30	8
Burry—Wales (Atlantic)	WBY	51°38.55′ N, 4°09.98′ W	8	8				8
Dundalk Bay—Ireland (Atlantic)	IDA	53°53.05′ N, 6°20.48′ W	8	8				8
Miño–Galicia Spain (Atlantic)	SMI	43°21.69′ N, 8°12.35′ W	30	30			30	
Ria de Noia—Spain (Atlantic)	SNO	42°47.42′ N, 8°55.36′ W	8	8				8
Campelo—Spain (Atlantic)	SCA	42°25.25′ N, 8°41.09′ W	30	30			30	
Redondela—Spain (Atlantic)	SRG	42°17.63′ N, 8°37.24′ W	30		30		30	19
Tejo Mouth–Portugal (Atlantic)	PTE	38°46.00′ N, 9°02.00′ W	8	8				8
Ria Formosa–Portugal (Atlantic)	PRF	36°59.86′ N, 7°49.81′ W	30	30			30	
Delta del Ebro—Spain (Mediterranean)	EDCE	40°41.75′ N, 0°45.12′ E	29		29			29
Nykobing Mors—Denmark (Atlantic) *	DNCE	56°46.91′ N, 8°52.27′ W	11	6	2	3		11
Espasante—Spain (Atlantic) *	EECE	43°43.44′ N, 7°48.26′ W	9	8		1		9
Carril—Spain (Atlantic) *	ECCE	42°36.75′ N, 8°46.50′ W	10	6	3	1		10
Combarro—Spain (Atlantic) *	EACE	42°25.92′ N, 8°42.30′ W	7	4		3		7
Lourizán—Spain (Atlantic) *	ELCE	42°24.53′ N, 8°40.66′ W	9	7		2		9

* Locations where suspected hybrids have been detected.

**Table 2:** External and internal primers (IP) for the two multiplex reactions developed in the present study. Lowercase letters in internal primers indicate the GACT nucleotide tails. Single nucleotide polymorphisms (SNPs) variants (with their position within their submitted GenBank sequence between parentheses), sense of the internal primer (IP sense; F: Forward, R: Reverse) and GenBank accession numbers for the sequences used to design the markers (length in bp of the sequence between parentheses) are shown.

Marker	SNPs Variants *C.edule*/*C. glaucum*	External Primers (5′-3′)	Tailed Internal Primer (5′-3′)	IP Sense	GenBank Accession Number
Cerast_1173A	A/G (129)	F: GGGACGGCACTTTTCACAAT R: TGGTGCTGTGGATGAATCGA	gactTGGTGGGCACTTGGATGC	R	MN178492 (240)
Cerast_1316	T/C (55)	F: TAGACAAAACAGGCCTACGC R: TCGTGATCTGCCAAAGGTTT	gactgactgacGCAACAGATTGCCAGCTGT	R	MN178494 (171)
Cerast_1400	G/A (99)	F: AGCACGGTTGTTGATTGGAC R: TTTAAGCCAGGGTCCTCGG	gactgactgactACTCTTCTTCATGGTTGAAAAGTC	F	MN178495 (218)
Cerast_456	C/T (90)	F: CAGCTTGGCATAACGTCACC R: TTATGCCTTGCGAATGTCCG	gactgactgactgactgactgaCAGAAGGATGCGGCATTGT	F	MN178490 (190)
Cerast_31	A/G (141)	F: CAGACCAGGCAAACACATCA R: CAGCTCGCATTTGTTCCCTT	gactgactgactgactgactgacATGATTAAGCAAGCTACTGCTAG	F	MN178488 (241)
Cerast_1255	A/T (115)	F: AATCGTTCATCATGTCCCGC R: AGTTGGTGTTCACAATTCCCC	gactgactgactgactgactgactgactgaATTGATTCGCAGTGTTTTGCT	R	MN178493 (192)
Cerast_586	C/T (154)	F: TGAATCTGTCCGCATCCTGA R: GATAAAACCTAACAGGTGGGC	gactgactgactgactgactgactgactgactgactgacATGGGCATGCGCAAAGG	F	MN178491 (213)
Cerast_450	A/T (165)	F: TTCACTCCACAACGAATCCA R: CCGGTACCCCAAACAATATAACA	gactgactgactgactgactgactgactgactgactgactCATTATAAATTCCTAGCGAGCAGA	F	MN178489 (250)
Cerast_2530	T/A (126)	F: TGTGATTTGTGTGGTGCTGT R: GTGTCTTCTAACATGGCATGCT	gactgactgactgactgactgactgactgactgactgactgactgactTGAATTTTGGCATGTTTTTGCTCTAG	R	MN178496 (243)

## Supplementary Materials

The following are available online at https://www.mdpi.com/2073-4425/10/10/760/s1, Table S1: Diagnostic single nucleotide polymorphisms (SNPs) for both cockle species analyzed in the present study. SNP position in the RADtag sequence (SNP position), SNP variants (Allele 1 and Allele 2), number of analyzed individuals (N), marker name (see main text and Table 2) and annotation against NCBI database are shown, Table S2: Private alleles detected for each cockle species. SNPs variants (Allele 1 and Allele 2), average number of reads per marker (Av. Nb reads), number of individuals analyzed (N), observed and expected heterozygosities (Ho and He, respectively), and inbreeding coefficients (Fis) are shown, Table S3: Genotypes obtained for the 96 individuals (48 *C. edule* and 48 *C. glaucum*) tested with the SNaPshot technique. Expected genotypes for each species with 2b–RAD–GBS technique and those previously found with this technique in control individuals are also shown. Morphological and molecular species identification is included for each individual. Bed codes are shown on Table 1, Table S4: Genotypes obtained with the SNaPshot technique for the 46 individuals from beds where the presence of hybrids has been suggested. For each individual, their identification using the methodology described by Freire et al. (2011) using ITS region (ITS) and the SNaPshot methodology described in the present study (SNaPshot) are shown. The 10 suspected hybrids and the multilocus genotypes for each species are shown in yellow and green, respectively. Bed codes are shown on Table 1.
